# Supplementary material for: Nutritional Status Associated with Molecular Biomarkers, Physiological Indices, and Clinical Severity in Parkinson’s Disease Patients
Source: Int J Environ Res Public Health. 2020 Aug 7;17(16):5727. doi: 10.3390/ijerph17165727 (PMC7459923; doi:10.3390/ijerph17165727)
Supplement: Supplementary file 1 [file ijerph-17-05727-s001.pdf]

Supplementary Materials: Nutritional Status Associated with Molecular Biomarkers, Physiological Indices, and Clinical Severity in Parkinson's Disease Patients

Tsu-Kng Lin [1,2,3,*], Yung-Yee Chang [1,2], Nai-Ching Chen [1], Chia-Wei Liou [1,2,3], Min-Yu Lan [1,2,3], Ying-Fa Chen [1,2] and Chia-Liang Tsai [4,*]

Table S1. The relationships between the mini nutritional assessment (MNA), the scores of Unified Parkinson's Disease Rating Scale (UPDRS), and the levels of biochemical markers in the PD patients and PD patients with malnutrition risk.

UPDRS	MNA	HgB	Cholesterol	HDL	LDL	HbA1c	BUN	Creatinine	Folate	VitB12
**Total participants**										
Section 1	*r* = –0.36; *p* = 0.001	*r* = 0.04; *p* = 0.730	*r* = –0.05; *p* = 0.671	*r* = –0.29; *p* = 0.009	*r* = 0.03; *p* = 0.765	*r* = –0.17; *p* = 0.134	*r* = 0.22; *p* = 0.054	*r* = 0.12; *p* = 0.297	*r* = 0.04; *p* = 0.729	*r* = 0.24; *p* = 0.033
Section 2	*r* = –0.35; *p* = 0.001	*r* = 0.03; *p* = 0.768	*r* = –0.02.; *p* = 0.877	*r* = –0.19; *p* = 0.092	*r* = 0.03; *p* = 0.783	*r* = –0.21; *p* = 0.069	*r* = 0.14; *p* = 0.233	*r* = –0.01; *p* = 0.902	*r* = 0.03; *p* = 0.778	*r* = 0.18; *p* = 0.108
Section 3	*r* = –0.18; *p* = 0.100	*r* = 0.07; *p* = 0.554	*r* = –0.20; *p* = 0.070	*r* = –0.13; *p* = 0.257	*r* = –0.16; *p* = 0.146	*r* = –0.16; *p* = 0.171	*r* = 0.28; *p* = 0.013	*r* = 0.10; *p* = 0.353	*r* = –0.00; *p* = 0.989	*r* = 0.17; *p* = 0.130
Section 4	*r* = –0.07; *p* = 0.542	*r* = –0.01; *p* = 0.945	*r* = 0.03; *p* = 0.825	*r* = –0.00; *p* = 0.996	*r* = 0.04; *p* = 0.733	*r* = –0.20; *p* = 0.077	*r* = –0.07; *p* = 0.514	*r* = –0.03; *p* = 0.807	*r* = –0.02; *p* = 0.850	*r* = –0.16; *p* = 0.179
Total scores	*r* = –0.29; *p* = 0.009	*r* = 0.06; *p* = 0.604	*r* = –0.14; *p* = 0.209	*r* = –0.18; *p* = 0.109	*r* = –0.09; *p* = 0.434	*r* = –0.21; *p* = 0.060	*r* = 0.24; *p* = 0.030	*r* = 0.07; *p* = 0.521	*r* = 0.01; *p* = 0.915	*r* = 0.19; *p* = 0.102
MNA	------	*r* = 0.25, *p* = 0.022	*r* = –0.13; *p* = 0.243	*r* = –0.16; *p* = 0.171	*r* = –0.08; *p* = 0.475	*r* = 0.23; *p* = 0.041	*r* = 0.05; *p* = 0.962	*r* = 0.05; *p* = 0.640	*r* = –0.02; *p* = 0.880	*r* = 0.07; *p* = 0.567
**Malnutrition risk**										
Section 1	*r* = –0.47; *p* = 0.020	*r* = –0.27; *p* = 0.211	*r* = 0.16; *p* = 0.452	*r* = –0.38; *p* = 0.066	*r* = 0.26; *p* = 0.219	*r* = –0.51; *p* = 0.16	*r* = 0.27; *p* = 0.207	*r* = –0.08; *p* = 0.702	*r* = 0.17; *p* = 0.467	*r* = 0.23; *p* = 0.309
Section 2	*r* = –0.47; *p* = 0.019	*r* = –0.01; *p* = 0.962	*r* = 0.10; *p* = 0.647	*r* = –0.19; *p* = 0.385	*r* = 0.14; *p* = 0.529	*r* = –0.41; *p* = 0.056	*r* = 0.08; *p* = 0.718	*r* = –0.04; *p* = 0.859	*r* = 0.17; *p* = 0.464	*r* = 0.27; *p* = 0.230
Section 3	*r* = –0.28; *p* = 0.189	*r* = 0.04; *p* = 0.838	*r* = –0.15; *p* = 0.496	*r* = –0.06; *p* = 0.798	*r* = –0.13; *p* = 0.532	*r* = –0.51; *p* = 0.016	*r* = 0.24; *p* = 0.270	*r* = 0.09; *p* = 0.687	*r* = 0.11; *p* = 0.619	*r* = 0.33; *p* = 0.145
Section 4	*r* = –0.08; *p* = 0.721	*r* = –0.13; *p* = 0.536	*r* = –0.03; *p* = 0.877	*r* = –0.09; *p* = 0.667	*r* = –0.04; *p* = 0.846	*r* = 0.18; *p* = 0.411	*r* = –0.14; *p* = 0.511	*r* = –0.08; *p* = 0.701	*r* = –0.25; *p* = 0.272	*r* = –0.24; *p* = 0.304

Total scores	*r* = –0.39; *p* = 0.058	*r* = –0.01; *p* = 0.948	*r* = –0.06; *p* = 0.798	*r* = –0.14; *p* = 0.505	*r* = –0.03; *p* = .900	*r* = –0.51; *p* = 0.016	*r* = 0.19; *p* = 0.375	*r* = 0.03; *p* = 0.894	*r* = 0.13; *p* = 0.579	*r* = 0.31; *p* = 0.170
MNA	------	*r* = 0.14, *p* = 0.527	*r* = 0.23; *p* = 0.278	*r* = 0.26; *p* = 0.221	*r* = 0.15; *p* = 0.498	*r* = 0.62; *p* = 0.002	*r* = –0.04; *p* = 0.849	*r* = 0.00; *p* = 0.998	*r* = 0.14; *p* = 0.534	*r* = 0.30; *p* = 0.188

PD: Parkinson's disease; UPDRS: Unified Parkinson's Disease Rating Scale; HgB: hemoglobin; HbA1c: glycated hemoglobin; MNA: mini nutritional assessment; HDL: high-density lipoprotein; LDL: low-density lipoprotein; BUN: blood urea nitrogen.
